# Sugar-Free Milk

**Published:** 1901-06-29

**Authors:** 


### SUGAR-FREE MILK.

Knowing as we do how markedly the glycosuria of diabetes is influenced by the administration of a sugar-free diet and one in which the carbohydrates are reduced to a minimum, and recognising how admirable a food milk would be for patients suffering from this disease but for the sugar which it contains, we cannot wonder that attempts should from time to time have been made with the object of producing a sugar-free milk for the use of diabetes. Dr. Robert Hutchinson[1] describes a process by which such a form of milk may be prepared, and the matter is one which we think is worthy of attention. The principle on which this process is founded is that out of one portion of milk the casein is abstracted, while out of another the fat is taken, and then the casein and fat so obtained are mixed together with water and made into "milk" again. The process is as follows:

Take a litre of skim milk, heat to a temperature of 38°C., and add 10cc. of glacial acetic acid diluted with 100cc. of water. Mix and allow the mixture to stand for about fifteen minutes. Collect the separated casein and let it drain on very fine muslin, using no pressure. Remove the casein to a mortar, rub into a smooth paste, add one half litre of distilled water, and strain as before. Repeat this washing of the casein twice. Transfer to a mortar, rub until quite smooth, and add 2·5 grammes of potassium hydrate dissolved in 100cc. of water (or as much of the KHO as is necessary to make the product just alkaline to phenolophthalein). Add 100 grammes of ordinary Devonshire clotted cream, 5 grammes of gelatin previously dissolved, ·06 gramme (1 grain) of saccharin and water at about 38°C. up to 1 litre. Lastly strain through fine muslin.

The product is said to taste very much like ordinary cow's milk, and can be taken either plain or with some effervescing water, or it can be added to tea or coffee or made into custard with eggs. In some cases as much as 5 pints of it has been taken in one day, and it does not seem to have any appreciable effect in increasing the output of sugar. Writing on the advantages of the use of this form of sugar-free milk, Dr. Hutchinson[2] says that it can hardly be distinguished from a rather rich specimen of ordinary milk. It also has the advantage that it can be made into boiled or even baked custards with eggs, thus rendering it possible to add a nourishing and harmless "sweet" course to the diabetic patient's dinner. This form of modified milk is of course specially useful in cases in which it is desirable to observe as strict a diet as possible, but even in those instances in which a limited amount of carbohydrate may be taken, it is found best to substitute this "sugar-free" milk for ordinary milk, and to give the allowable quantity of carbohydrate in other forms, such as bread or potatoes. Not only is this plan more satisfactory to the patient, as giving him the variety he craves for, but it also has the great advantage of enabling one to administer larger quantities of butter. Dr. Hutchinson gives the following as a strict diet in which this form of milk is used:—

*Breakfast.*—Bacon; eggs scrambled, with butter; fish and butter sauce or some form of cold meat; toasted protene bread with plenty of butter; and café-au-lait made with sugar-free milk and sweetened with saccharine if desired.

*Dinner.*—Soup (without vegetables or other carbohydrate-containing matter); fish (preferably one of the fatter sorts); meat with green vegetables and melted butter; custard of eggs and sugar-free milk; and cheese with protene bread and watercress. Beverage: whiskey-and-water or any dry natural wine.

*Supper.*—A cup of well-made beef tea; eggs in some form—*e.g.*, as an omelette with as much butter as possible; fish or cold meat; and cheese with protene bread-and-butter and a salad with plenty of salad oil. Beverage: a glass of sugar-free milk.

The rest of the milk, sufficient to make the total daily allowance up to three pints, should be taken as a beverage between the chief meals.

The matter is one of considerable interest, for anything which will enable a diabetic patient to have the run of milk and milk puddings will vastly lessen the difficulty of making him contented with his diet. We should think, however, although we have not tried it, that a good deal of the more troublesome part of Mr. Hutchinson's plan might be saved by the use of plasmon. All the first portion of Dr. Hutchinson's process is gone through for the sake of getting a soluble casein, and this is just what plasmon is, so that the presumption is that if the proper proportions of plasmon, Devonshire cream, dissolved gelatin, saccharin, and water were carefully mixed as above described, a very analogous product would be obtained with far less trouble.

[1] Food and the Principles of Dietetics. [2] Lancet, June 22.

